# Spatiotemporal access to emergency medical services in Wuhan, China: accounting for scene and transport time intervals

**DOI:** 10.1186/s12942-020-00249-7

**Published:** 2020-11-26

**Authors:** Weicong Luo, Jing Yao, Richard Mitchell, Xiaoxiang Zhang

**Affiliations:** 1 Centre for Sustainable, Healthy and Learning Cities and Neighbourhoods, University of Glasgow, Glasgow, UK; 2 Urban Big Data Centre, School of Social & Political Sciences, University of Glasgow, 7 Lilybank Gardens, Glasgow, G12 8RZ UK; 3 MRC/CSO Social & Public Health Sciences Unit, University of Glasgow, Glasgow, UK; 4 Department of Geographic Information Science, College of Hydrology and Water Resources, Hohai University, Nanjing, China

**Keywords:** Spatiotemporal access, EMS, GIS, Online map services, E-2SFCA

## Abstract

**Background:**

Access as a primary indicator of Emergency Medical Service (EMS) efficiency has been widely studied over the last few decades. Most previous studies considered one-way trips, either getting ambulances to patients or transporting patients to hospitals. This research assesses spatiotemporal access to EMS at the *shequ* (the smallest administrative unit) level in Wuhan, China, attempting to fill a gap in literature by considering and comparing both trips in the evaluation of EMS access.

**Methods:**

Two spatiotemporal access measures are adopted here: the proximity-based travel time obtained from online map services and the enhanced two-step floating catchment area (E-2SFCA) which is a gravity-based model. First, the travel time is calculated for the two trips involved in one EMS journey: one is from the nearest EMS station to the scene (i.e. scene time interval (STI)) and the other is from the scene to the nearest hospital (i.e. transport time interval (TTI)). Then, the predicted travel time is incorporated into the E-2SFCA model to calculate the access measure considering the availability of the service provider as well as the population in need. For both access measures, the calculation is implemented for peak hours and off-peak hours.

**Results:**

Both methods showed a marked decrease in EMS access during peak traffic hours, and differences in spatial patterns of ambulance and hospital access. About 73.9% of shequs can receive an ambulance or get to the nearest hospital within 10 min during off-peak periods, and this proportion decreases to about 45.5% for peak periods. Most shequs with good ambulance access but poor hospital access are in the south of the study area. In general, the central areas have better ambulance, hospital and overall access than peripheral areas, particularly during off-peak periods.

**Conclusions:**

In addition to the impact of peak traffic periods on EMS access, we found that good ambulance access does not necessarily guarantee good hospital access nor the overall access, and vice versa.

## Background

Emergency Medical Services (EMS) provide medical care to patients with serious illnesses or injuries that require rapid response, offering prehospital medical treatment and transferring them to hospital. In addition to responding to medical emergencies, EMS plays a critical role in the rescue activities involved in all kinds of accidents and disasters, protecting people’s health and safety [25]. An accessible EMS system is crucial to high service quality and favourable health outcomes [2, 15, 21, 24]. Understanding access to EMS can help healthcare planners to improve health resource deployment and service efficiency.

Access to health services is a multi-dimensional concept, which has been extensively studied by scholars from a range of disciplines including public policy, geography and sociology [6, 13, 18, 32]. From the utilization perspective, access can be differentiated between potential and revealed access [18], where the former refers to the chance of using health services and the latter represents the actual utilization of health services, that is, the achievement of potential access. From a spatial perspective, geographic access often refers to the travel impedance (e.g. travel distance or travel time) between health facilities (e.g. hospitals or clinics) and patients [18]. The non-spatial perspective primarily concerns about factors like demographic characteristics, socioeconomic status and health insurance schemes that could affect the easiness of healthcare acquisition [9]. Of interest in this research is the potential geographic access to EMS and its variation over space.

Numerous measures of potential geographic access to healthcare have been developed over the last few decades, which can be categorized as provider-to-population ratios, distance-based measures, and gravity-based models [4, 12, 16, 32]. Provider-to-population ratios are often calculated using data aggregated at certain spatial scales such as administrative units or catchment area of health facilities. Common distance-based measures include travel distance and time, where the former is often represented by Euclidean or road network distance between a patient residence and the nearest healthcare provider, and the latter is the time required to complete the journey for seeking healthcare by certain transport modes like walking or driving. Gravity-based models incorporate the above two methods and account for the interactions between health services and potential demand, which generally follows a distance-decay effect. Since the pioneering work by Joseph and Bantock [18], two most well-known and established gravity-based models are the two-step floating catchment area (2SFCA) [23] and one of its extensions, namely enhanced 2SFCA (E-2SFCA) [22]. All three aforementioned types of methods have been widely adopted to measure healthcare access in a variety of contexts (e.g. [5, 7, 17, 38]).

**Fig. 1:**
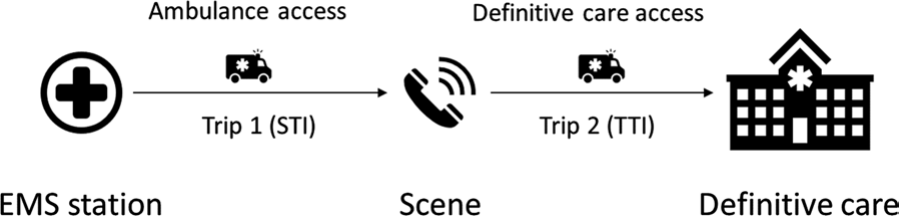
Common procedure of EMS

With respect to EMS, the response time obviously is critical and can greatly affect the chance of survival. Furthermore, EMS involves two related trips (Fig. 1): the first from the EMS station to the scene (hereafter referred to as trip 1) and the second from the scene to the care facility, hospital or trauma center (hereafter referred to as trip 2). Trip 1 plays a vital role in saving lives and trip 2 is equally important. Ambulances can only provide basic medical assistance, and patients often need further treatment. For a complete EMS journey, the response time generally consists of scene time interval (STI), patient access time interval (PATI), on-scene time interval (OSTI), and transport time interval (TTI) [28]. STI and TTI are directly associated with the travel impedance for trip 1 and trip2, respectively, as shown in Fig. 1. PATI is the time interval between vehicle arrival at scene and hands on patient, and OSTI is the time interval between arrival at patient and beginning moving patient. Similar to STI and TTI, PATI and OSTI are known to significantly impact health outcomes [10, 11, 19, 26]. The total travel time for trip 1 and trip 2, represented by the sum of SIT and TTI, is clearly related to geographic proximity (both of ambulance to patient and then patient to hospital), but will also depend on the road quality, real-time traffic and weather conditions among other factors.

One limitation in the studies on EMS access is that the travel time involved in distance and gravity-based approaches is often estimated by assuming a constant travel speed for particular land-use types (e.g. [8]), road classes (e.g. [5]) and transportation modes (e.g. [20]), or by using transportation simulation models (e.g. [17]) that fail to capture the variations in travel time resulting from real traffic conditions which undoubtedly is crucial to EMS. Whilst distance is constant, the time taken to cover it is clearly and heavily influenced by traffic levels and speed which have extreme diurnal variation. Although some countries have a culture whereby traffic gives way to an ambulance, not all do. This is a particular problem in China where there are regular incidents in which ambulances are not allowed to pass through traffic by other drivers. Nowadays, travel time can be readily predicted through online map services such as Google Maps (https://www.google.com/maps) and many mobile applications such as TripAdvisor and TripIt provide travel planning services. Recently, such services and applications have been increasingly employed in measuring healthcare access [30, 33], which may offer more accurate travel time estimation based on real-time traffic. Attracted by these features, we adopt online map services in this research to estimate the travel time involved in the spatiotemporal access to EMS.

Another limitation of existing healthcare access measure is that in general, only one-way trips–from the home to the health facility–are considered. Although this is usually the case for general healthcare-seeking behaviour, it is not applicable to EMS, which involves the two related trips (Fig. 1). The hospital or care facility may not be at the same location as the EMS station is. EMS teams also regularly bypass local hospitals to transfer the patient to the facility that is most adapted to their condition (angioplasty, trauma center, stroke unit, etc.). Some patients might be reached easily and quickly by an ambulance but have a long journey to the care locations, or vice versa. Existing EMS access measures generally consider either trip 1 (e.g. [17]) or trip 2 (e.g. [36]). Although Vanderschuren and McKune [31] considered both trips in measuring access of road fatalities to emergency care facilities, they employed static road network with speed limits. Given the importance of both trips to survival rates, one objective of this research is to fill a gap in literature by considering and comparing both trips (i.e. STI and TTI) in the evaluation of EMS access with online map services. Like any other health services, access to EMS is also subject to the availability of resources like ambulances and medical staff as well as population in need. In addition, the hospital capacity is critical as well from the planning perspective, which can affect EMS access particularly in the outbreak of major accidents, hazards (e.g. earthquakes) or pandemics like COVID-19. Therefore, the other objective of this research is to combine online map services and E-2SFCA to evaluate spatiotemporal access to EMS.

This study assesses spatiotemporal access to EMS in Wuhan, China. Specifically, the aim of this research is threefold: to measure spatiotemporal access to EMS (1) based on predicted travel time with online map services; (2) based on E-2SFCA accounting for the interaction of healthcare demand and provider; (3) to compare the EMS access between trip 1, trip 2 and both trips. Wuhan is the largest city as well as the hub of economy, logistics, culture, education, transportation and commerce in Central China. It was the epicenter of COVID-19 in late 2019 and early 2020. In this difficult period, EMS played a significant role by responding to emergency calls and transporting patients for timely treatment. Understanding access to EMS can help identify the areas that underserved and thereby inform policy makers regarding healthcare planning and resource allocation.

## Methods

### Study area and data

The study area is Wuhan, the capital city of Hubei Province and the largest city in Central China. It is located on the intersection of the Yangtze River and the Han River, covering an area of 8,569.2 km^2^ with a population 11.2 million by the end of 2019 [35]. Wuhan nowadays is the central city of the urban agglomeration in the middle reaches of the Yangtze River and is one of the nine National Central Cities of China, leading the rise of Central China [29]. In recent years, Wuhan has attracted urban and rural migrants from across the country given its rapid urban development and economic achievements, which has raised the demand for public services such as healthcare, housing and transportation.

Wuhan includes 13 urban districts: 7 in the central urban area and 6 in the suburban and rural area. The targets for the EMS response time are different for the central urban districts and the suburbs: 10 and 15 min, respectively [34]. Considering that traffic congestions, which can greatly affect the travel time of ambulances [1], are more likely occur in the city center, we restricted our analysis to the seven central urban districts. Residences are adopted here to represent the scenes (i.e. patient origins), which is a common option in assessing potential spatial access to EMS in the lack of real EMS run data [2, 17, 33]. The data used in this research consist of 1,172 local communities (often called *Shequ* in China), 41 EMS stations and 53 definitive care facilities (equivalent to hospitals in Wuhan). Shequ is the smallest administrative unit in China that covers a certain geographic area where people have close social interaction; it is also the finest spatial scale at which the census population data are available. In our dataset, the average population per shequ is 5490. No readily available dataset described the number of ambulances based at each EMS station in Wuhan. We therefore assume that all EMS stations have the same number of ambulances (usually two to three). Hospitals in China are grouped into three categories: Grade I, II or III. Grade III hospitals have the highest medical capacity. In our study, only Grade II and Grade III hospitals are included, as they are the main definitive care facilities in China. The population of each shequ represents the demand, and EMS stations and hospitals are the service providers.

All data used are from open sources and freely available from the data providers. The location information was obtained from Baidu Map (https://map.baidu.com/), which is the largest online map service provider in China. There are 17 locations that contains both an EMS station and a hospital. Thus, there are total 77 unique locations of EMS stations or hospitals. The populations of the shequ and hospital attribute data were derived from the Geographical Information Monitoring Could Platform (http://www.dsac.cn/), which is the most used online resource for geographic, natural resource, environmental, climate and socioeconomic data in China.

**Fig. 2:**
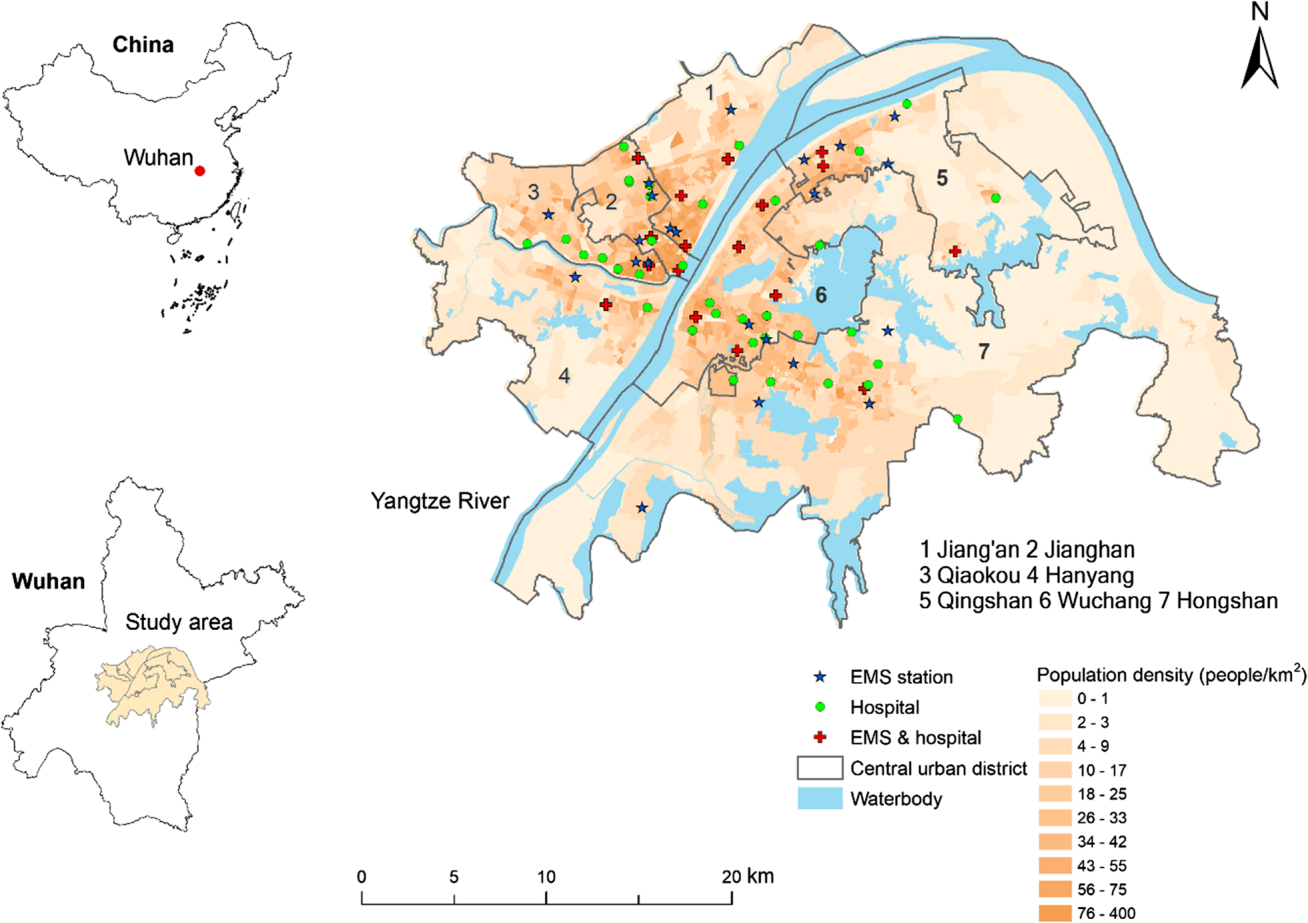
Study area and population density in central urban districts in Wuhan

Figure 2 shows the population density of each shequ across the study area grouped by quartile classification. About 30% of shequ have density of less than 10 people/km^2^, and such shequ are predominantly located in the periphery of the study area. About 30% of Shequ have a population density higher than 43 people/km^2^, and they are mainly concentrated in the central part of the study area on both sides of the Yangtze River. In general, all the districts to the west of the Yangtze River, except Hanyang, are densely populated whereas to the east of the river, only Wuchang has a relatively higher population density. In particular, Jianghan has the highest population density while Hongshan has the lowest. Figure 2 also shows the locations of the EMS stations and hospitals, which are mainly situated in areas with high population density.

### Measuring Spatiotemporal Access to EMS

**Fig. 3:**
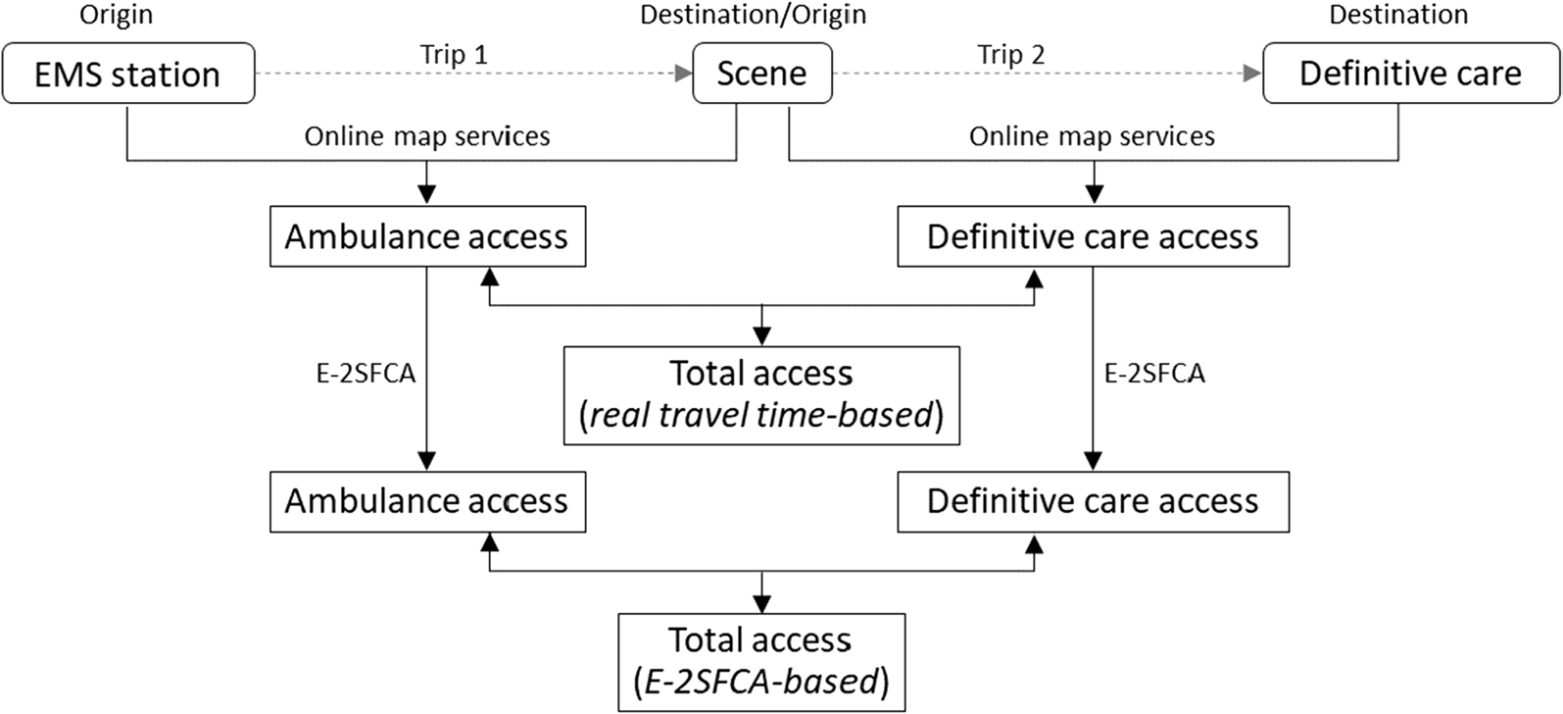
Analysis Framework of Spatiotemporal Access to EMS

Due to the lack of real EMS run data, the potential EMS access considered in this research will focus on SIT of trip 1 and TTI of trip 2, which are commonly employed in the estimation of potential EMS access [17, 36]. Two spatiotemporal access measures are adopted here: one is the proximity-based travel time obtained from online map services, and the other is the gravity-based model E-2SFCA. The analysis framework is presented in Fig. 3. First, travel time is estimated for the two trips involved in one EMS journey: the first trip is from the nearest EMS station to the scene assuming an ambulance is always available at the nearest station (i.e. STI), and the other is from the scene to the nearest hospital that provides definitive care (i.e. TTI), where the scene is represented by the centroid of each Shequ. Then, STI and TTI are incorporated into the E-2SFCA model to calculate the access measure considering the availability of the service provider as well as the population in need. For both access measures, the calculation is implemented for peak hours and off-peak hours. We considered two peak time windows (7:40–8:00 and 18:00–18:20) and three off-peak time windows (10:00–10:20, 15:00–15:20 and 21:00–21:20) in this study based on the peak/off-peak periods specified by Wuhan Traffic Management Bureau.

The travel time was calculated using the Baidu Map online service, which has the most updated road network in China and accounts for real traffic flows. Specifically, Java scripts were developed to call Baidu Map’s Application Programming Interface (API) through the open platform of Baidu Map for route planning (https://lbsyun.baidu.com/products/products/direction). Considering that the impact of traffic congestion occurs mainly during weekdays, we calculated travel time for the five days (Monday to Friday) between September 23–27, 2019, when the weather in Wuhan was good and there were no big events that would affect traffic. As a result, there are total 58,600 trips (two trips for each Shequ on every weekday at each period) are analysed in this study.

The 2SFCA consists of two steps. The first step is to calculate the provider-to-population or supply-to-demand ratio within a certain travel time of each health facility (i.e. EMS station or hospital). The second step is to sum up the provider-to-populations ratios of all the health facilities within the pre-defined travel distance/time of each population location, which is therefore a population-based access measure. The E-2SFCA [22] adapts the 2SFCA by dividing the overall travel into several time zones, each with an associated weight that follows a distance-decay effect. The E-2SFCA is employed here because EMS often has a required minimum response time, such as 10 or 15 min, which can be utilized to define different time zones.

Using the following notation:

*i*, *j*, *k*: the index of EMS stations, hospitals and scenes (population locations), respectively;

${E}_{i}$, ${H}_{j}$: the supply capacity at the $i$ th EMS station and the $j$ th hospital, respectively;

${P}_{k}$: the population at location $k$;

${t}_{ik}$, ${t}_{jk}$: the travel time from the $i$ th EMS station to the $k$ th population location, and from the $k$ th population location to the $j$ th hospital, respectively;

$r$, ${T}_{r}$, ${w}_{r}$: the index of travel time zones, the $r$ th time zone and its associated weight, respectively.

The definition of E-2SFCA in the context of this research can be formulated as in (1, 2, 3):

1 $$R_{i} = \frac{{E_{i} }}{{\sum _{r} \sum _{{k \in (t_{{ik}} \in T_{r} )}} P_{k} w_{r} }} \quad R_{j} = \frac{{H_{j} }}{{\sum _{r} \sum _{{k \in (t_{{jk}} \in T_{r} )}} P_{k} w_{r} }}$$

2 $${A}_{k}^{E}={\sum }_{r}{\sum }_{i\in ({t}_{ik\in {T}_{r}})}{R}_{i}{w}_{r} \quad {A}_{k}^{H}={\sum }_{r}{\sum }_{j\in ({t}_{jk\in {T}_{r}})}{R}_{j}{w}_{r}$$

3 $${A}_{k}={A}_{k}^{E}+{A}_{k}^{H}$$

Therefore, the provider-to-population ratio for the $i$ th EMS station and the $j$ th hospital, denoted by ${R}_{i}$ and ${R}_{j}$, respectively, are calculated first. Then, the access measure of the $k$ th population location in relation to EMS stations and hospitals, denoted by ${A}_{k}^{E}$ and ${A}_{k}^{H}$, respectively, are derived by summing up the corresponding weighted ${R}_{i}$ and ${R}_{j}.$ Finally, the overall access ${A}_{k}$ is defined as the sum of ${A}_{i}^{E}$ and ${A}_{i}^{H}$, with higher values indicating better access.

Regarding the values of the parameters in (1, 2, 3), all the EMS stations are assumed to have the same number of ambulances due to the lack of data. That is, ${E}_{i}=2$ for all $i$. ${H}_{j}$ is defined as the number of inpatient beds in each hospital, ranging from 50 to 3300. As two EMS response times are adopted in Wuhan: 10 min for the central urban districts and 15 min for the suburbs, three time zones are adopted in this research. That is, $r\in \{\mathrm{1,2},3\}$. The value of ${w}_{r}$ is then determined based on those three time zones. Specifically, if ${t}_{ik}$ or ${t}_{jk}$ is less than 10 min, ${w}_{r}=1$; if ${t}_{ik}$ or ${t}_{jk}$ exceeds 10 min but is less than 15 min, the value of ${w}_{r}$ decays with the increase of ${t}_{ik}$ or ${t}_{jk}$, and is calculated using the Gaussian function that is commonly applied in healthcare access studies (see [7],if ${t}_{ik}$ or ${t}_{jk}$ is more than 15 min, ${w}_{r}=0$. Finally, considering the different scales of ${E}_{i}$ and ${H}_{j}$ values used here, ${A}_{k}^{E}$ and ${A}_{k}^{H}$ are standardized using Eq. (4) before being applied to Eq. (3) to obtain ${A}_{k}$. In Eq. (4), $v$ represents ${A}_{k}^{E}$ or ${A}_{k}^{H}$, and ${v}^{^{\prime}}$ is the standardized value. Thus, the values of both ${A}_{k}^{E}$ and ${A}_{k}^{H}$ have a range of 0 to 1, which are relative measures representing the ‘relative access’ in comparison with the minimum (i.e. poorest access) and the maximum scores (i.e. best access). Specifically, a Shequ has the poorest access within the study area if it has a score 0, the best access if it has a score 1. For the Shequs with a score between 0 and 1, higher values indicate relatively better access.

4 $${v}^{^{\prime}}=(v-{v}_{min})/({v}_{max}-{v}_{min})$$

## Results

### Travel time-based spatiotemporal access to EMS

**Fig. 4:**
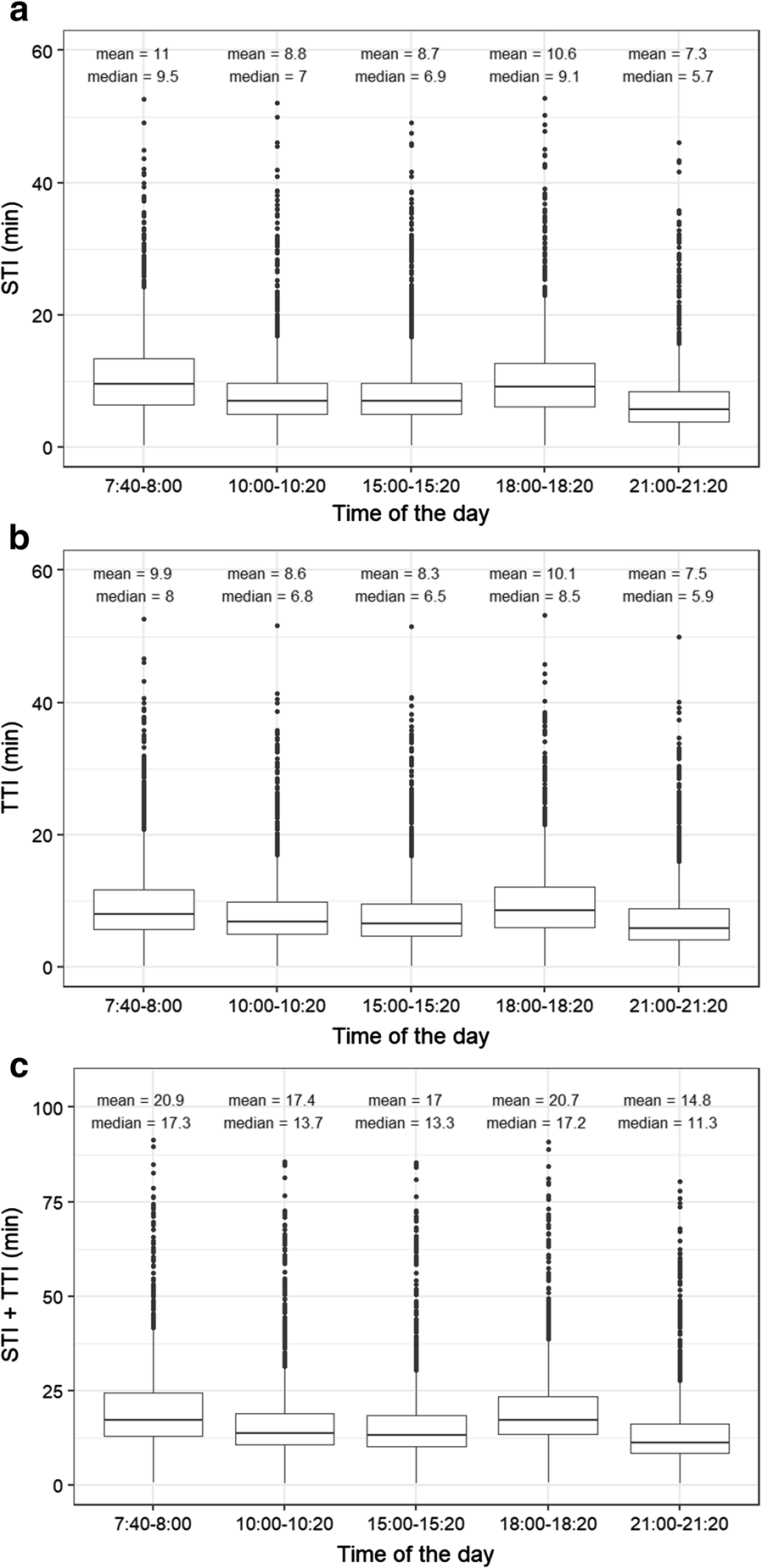
Boxplots of predicted travel time for different times of the day: **a** trip 1 (EMS station → Scene); **b** trip 2 (Scene → Hospital); **c** total trip (EMS station → Scene → Hospital)

For each time period of the day, travel times on the five weekdays were averaged for each shequ. Figure 4 shows the distribution of the predicted travel time for the three trips: trip 1 (EMS station Scene), trip 2(Scene Hospital) and total trip (EMS station Scene Hospital). Figure 4 shows that the distributions of STI and TTI for the two peak hour periods (i.e. 7:40–8:00 and 18:00–18:20) are very similar and are clearly different from those for the three off-peak periods. The peak hour travel times have higher mean and median values as well as larger interquartile ranges. According to the median travel time shown in Fig. 4a, b, over 50% of shequs can be reached by an ambulance from the nearest EMS stations within 10 min, and again a patient can be transported from his/her shequ to the nearest hospital within 9 min. On average, it takes longer for the nearest ambulance to get *to* a shequ than to travel to the nearest hospital *from* a shequ during all but evening peak periods. Figure 4c indicates that the total journey takes nor more than 18 min for more than half of the shequs.

During peak periods, for over 75% of shequs, it takes less than 14 min to get an ambulance and less than 13 min to get to the nearest hospital, and the total journey takes less than 25 min. For trip 1 (Fig. 4a) and the total trip (Fig. 4c), the average travel time during morning peak hours is longer than that in the evening peak hours, while the opposite is true for trip 2 (Fig. 4b). During off-peak periods, over 75% of shequs can be reached by the nearest ambulance within 10 min, which can get to the nearest hospital within another 10 min, with total trip taking less than 19 min.

It is not surprising that the travel time is longer during peak periods than that in off-peak periods for the same journey. For example, on average, it takes 11.0 min and 10.6 min from the nearest EMS station to a shequ during the morning and evening rush hours, respectively, but it only takes about 7.3–8.8 min for the same journey during off-peak periods, as shown in Fig. 4a. Figure 4c indicates that the average travel time for total trip during peak periods is nearly 21 min but 3–6 min less in off-peak periods. Among all time periods of the day, the evening off-peak hours (21:00–21:20) have the least travel time for all three trips. It also should be noted that for some shequs, it can take much longer than the average travel time to receive an ambulance or get to the nearest hospital. For example, it can take up to 53 min for an ambulance to arrive at a shequ (Fig. 4a) or get to the nearest hospital from a shequ in the morning peak hours (Fig. 4b). Even in the evening off-peak hours, it can take up to 80 min for a complete trip (Fig. 4c).

**Fig. 5:**
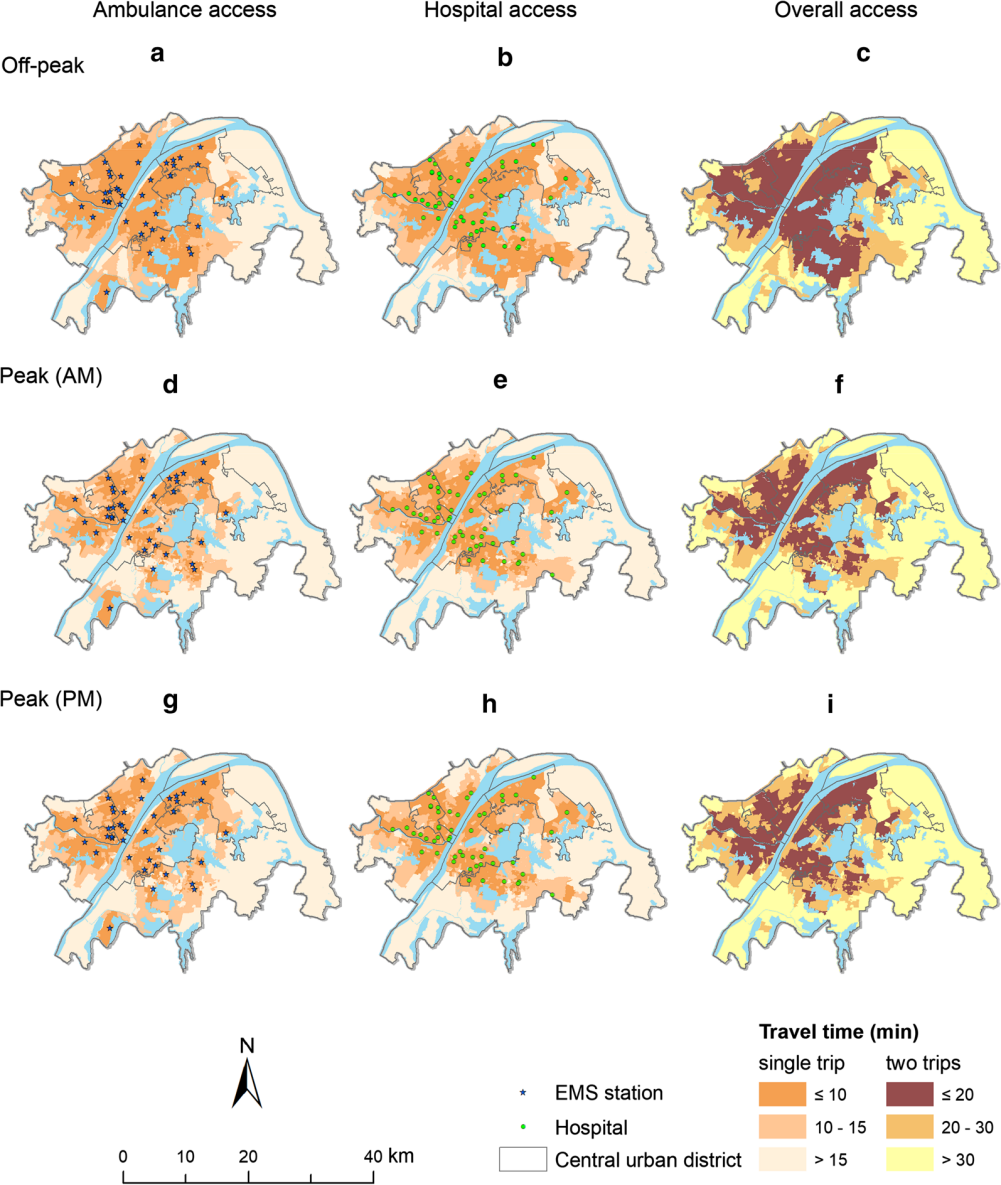
Average travel time for single and total trips at different times of the day (Off-peak periods: **a** ambulance access, **b** hospital access, **c** overall access; Morning peak periods: **d** ambulance access, **e** hospital access, **f** overall access; Evening peak periods: **g** ambulance access, **h** hospital access, **i** overall access)

The travel times in the three off-peak periods were further averaged for each shequ. The spatial variations of the average travel times for the three time periods of the day (i.e., off-peak, morning peak and evening peak) are depicted by Fig. 5. Most shequs with good ambulance and hospital access (i.e. STI or TTI ≤ 10 min) as well as overall access (i.e. STI + TTI ≤ 20 min) are concentrated in the central part of the study area. In particular, Lianhe shequ in Jiang’an district has the best ambulance access and overall access, and Hongwei shequ in Qingshan district has the best hospital access. In contrast, most shequs with relatively poorer access (i.e. STI or TTI > 15 min or STI + TTI > 30 min) are located in the periphery of the study area. Particularly, the three shequs in Hongshan district, Kuailing, Yangling and Xiejia, have the poorest ambulance, hospital and overall access, respectively. Also, fewer Shequs can receive an ambulance or get to the nearest hospital within 10 min (Fig. 5d, e, g, h) or complete the total trip within 20 min (Fig. 5f, i) during peak periods compared with the off-peak period (Fig.  5a–c). For the same Shequ, STI and TTI can be different. For example, some shequs in Hongshan district that are close to the only EMS station in the southwest of the study area (see Fig. 2) can get an ambulance within 10 min during all times of the day (Fig. 5a, d, g), but it takes more than 15 min from the same shequ to the nearest hospital (Fig. 5b, e, h).

Table 1 summarizes the percent of shequs in each travel time group during peak and off-peak periods. Similar to Fig. 5, Table 1 suggests that traffic congestion during peak hours has a marked impact on the travel time. For example, most shequs (about 73.9%) can receive an ambulance or get to the nearest hospital within 10 min during off-peak periods. This proportion decreases to 45.5% and 45.9% for the two peak periods. Only 8.3% of the shequs need to wait more than 15 min to get an ambulance or carry a patient to the nearest hospital during off-peak periods, but the proportion increases to 12.2% and 11.5% during the morning and evening peak periods, respectively.

**Table 1:** Proportion of shequs in each travel time group (off-peak, peak (AM), peak (PM))

	Trip 2 travel time			
		$\le$ 10 min	10–15 min	> 15 min
Trip 1 travel time	$\le$ 10 min	(73.9, 45.5, 45.9)	(4.3, 6.7, 10.2)	(0.3, 0.9, 1.3)
	10–15 min	(4.4, 15.8, 14.1)	(5.0, 9.0, 7.8)	(1.5, 2.0, 3.1)
	> 15 min	(0.8, 3.8, 2.8)	(1.5, 4.1, 3.3)	(8.3, 12.2, 11.5)

**Fig. 6:**
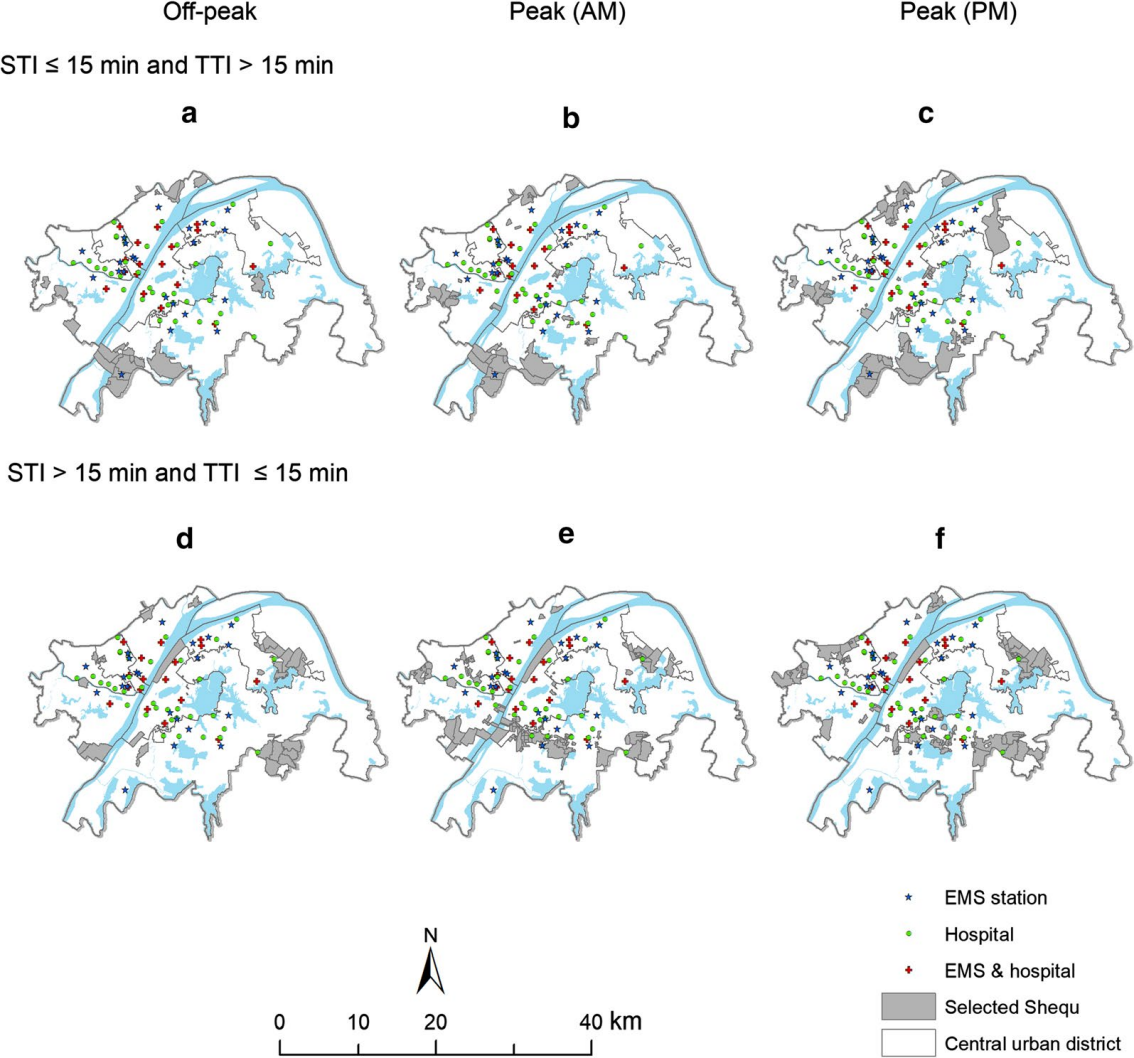
Locations of the Shequ within different travel time groups for STI and TTI (STI $\le$ 15 min and TTI > 15 min: **a** off-peak periods, **b** morning peak periods, **c** evening peak periods; STI > 15 min and TTI $\le$ 15 min: **d** off-peak periods, **e** morning peak periods, **f** evening peak periods)

Figure 6 highlights the shequs (in grey) within different travel time groups for STI and TTI. Specifically, the highlighted shequs have STI less than or equal to 15 min and TTI larger than 15 min, and vice versa. Figure 6a–c indicate that most shequs, which can be reached by an ambulance within 15 min but are more than 15 min away from the nearest hospital, are largely in the northern and southern periphery of the study area. Comparatively, most shequs, for which it takes more than 15 min for the nearest ambulance to arrive but no more than 15 min to get to the nearest hospital, are closer to the central areas, with a few scattered in the northern and western periphery of the study area (see Fig. 6d–f). Also can be observed is that the number of highlighted shequs increases if the trips occur during peak periods. In the case where only STI is within 15 min (Fig. 6a–c), the shequs affected by peak periods are mainly located in Jiang’an, Hangyang, Qingshan and Hongshan districts. In comparison, there are more affected shequs when only TTI is within 15 min (Fig. 6d–f), and there are primarily located in Hangyang, Jianghan and Hongshan districts.

### E-2SFCA-based spatiotemporal access to EMS

**Fig. 7:**
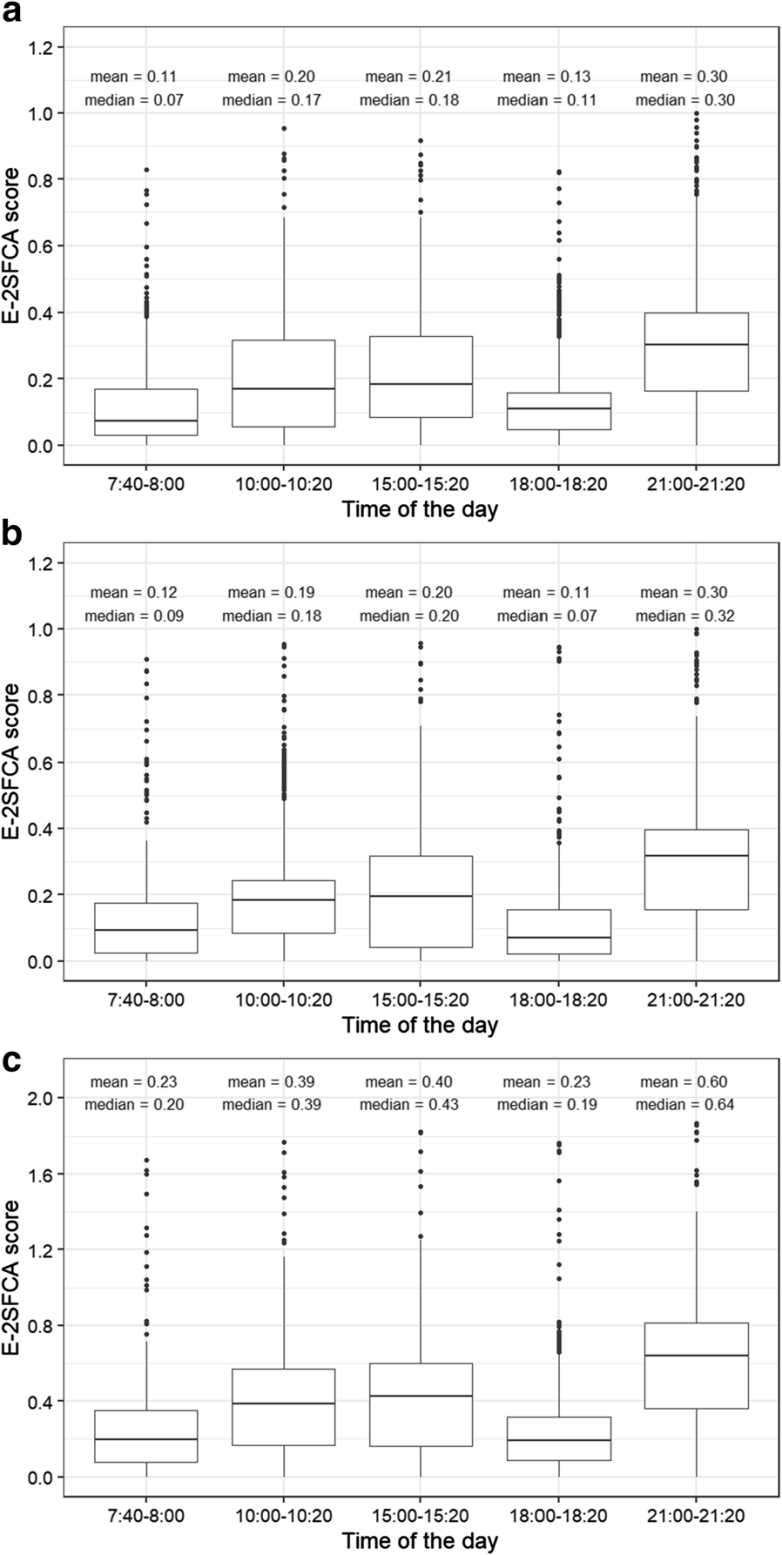
Boxplots of E-2SFCA access scores for different times of the day: **a** trip 1 (EMS station Scene); **b** trip 2 (Scene Hospital); **c** total trip (EMS station Scene Hospital)

The variations of standardized E-2SFCA access scores for different times of the day are presented in Fig. 7. Given the range of values (i.e. 0–1), most of the scores are relatively low, with over 75% of shequs having a value lower than 0.4 for single trip and 0.8 for the total trip. Most average scores for the different periods are less than 0.2 for a single trip and 0.4 for the total trip. Among the five periods, the evening off-peak period seems to generate the highest score, indicating the best access. Similar to the travel time in Fig. 5, there are clear differences between the results of the peak and off-peak periods. For example, over 75% of shequs have a score lower than 0.2 for both trip 1 and trip 2 during peak periods, while this proportion reduces to below 50% for off-peak periods. Comparing the two peak periods, access in the morning peak hours seems better than that in the evening peak hours for trip 2 (Fig. 7b) and the total trip (Fig. 7c) but worse for trip 1 (Fig. 7a). Also, it can be observed that there are more variations (a larger quartile) in the access scores for the morning off-peak periods in trip 1 (Fig. 7a) than in trip 2 (Fig. 7b).

**Fig. 8:**
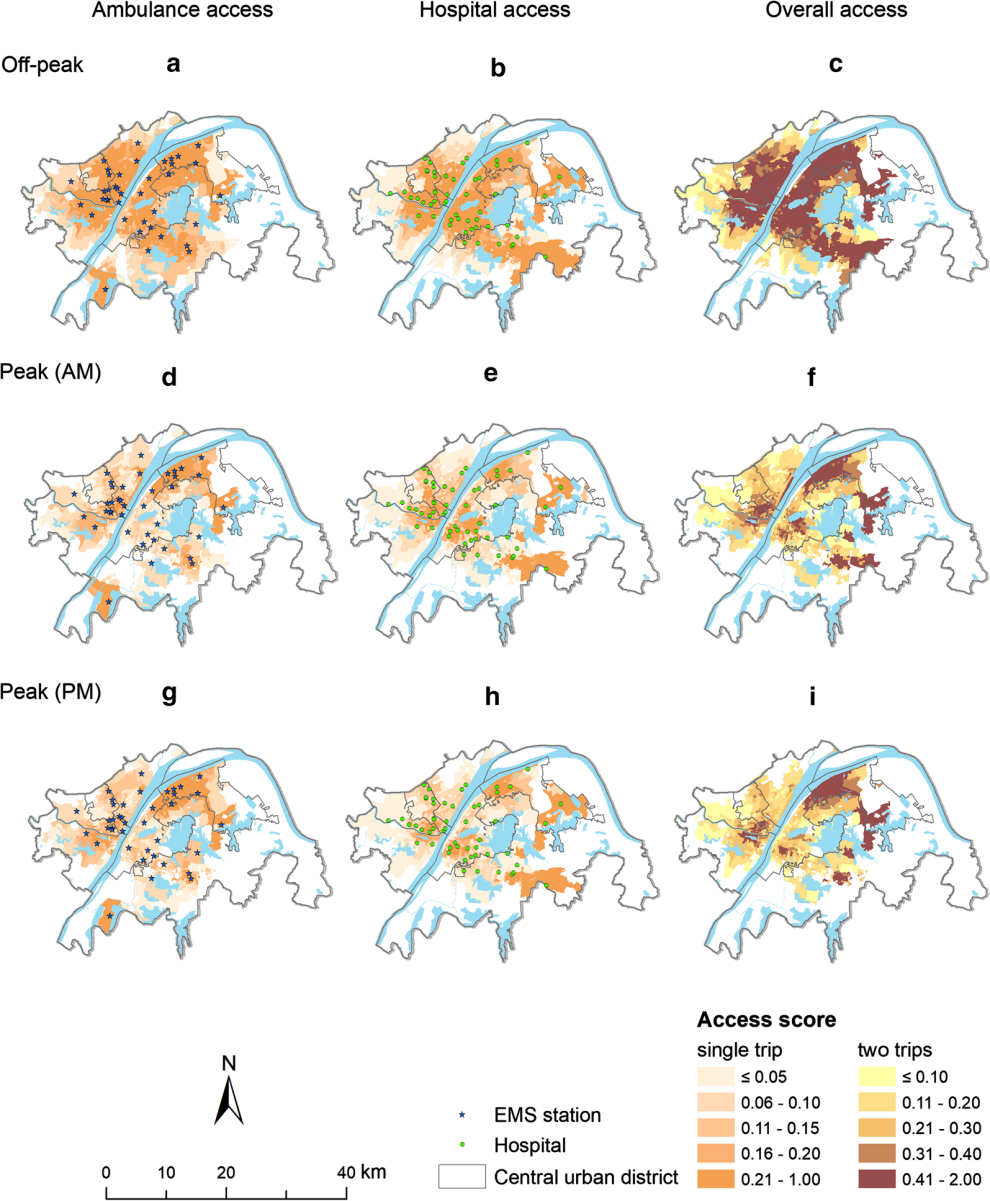
Average E-2SFCA access score for single and total trips at different times of the day (Off-peak periods: **a** ambulance access, **b** hospital access, **c** overall access; Morning peak periods: **d** ambulance access, **e** hospital access, **f** overall access; Evening peak periods: **g** ambulance access, **h** hospital access, **i** overall access)

Figure 8 depicts the spatial variations of the average E-2SFCA access scores for the single and total trips during different times of the day, where the shequs having travel time longer than 15 min for both trips are left blank (i.e. ${A}_{i}^{E}$ = 0, ${A}_{i}^{H}$ = 0 or ${A}_{i}$ = 0). Considering the distribution of the E-2SFCA scores in Fig. 7, the E-2SFCA scores higher than 0.2 are considered here representing “relatively good access” and the classification in Fig. 8 is used to clearly describe the spatial variations of E-2SFCA based access. Similar to the pattern observed in Fig. 5, the central areas have better ambulance, hospital and overall access than peripheral areas, particularly during off-peak periods. Again, Lianhe shequ in Jiang’an district has the highest ${A}_{i}^{E}$ and ${A}_{i}$ scores in the off-peak periods, indicating best ambulance access and overall access. Jianqiao shequ in Hongshan district has the best hospital access. When comparing access between the off-peak and peak periods, it can be seen that the access score for all three types of access (ambulance, hospital and overall) in general decreases for most of the shequs during peak periods. In other words, only some shequs along the Yangtze River and a few in Qingshan and Hongshan have consistently good access (i.e. ${A}_{i}^{E}$ and ${A}_{i}^{H}$ > 0.2 and ${A}_{i}$ > 0.4) for a single trip and the total trip during any time of the day. The shequs to the east of Yangtze River and within Wuchang are most affected by the traffic during rush hours with evident decrease in access scores.

**Fig. 9:**
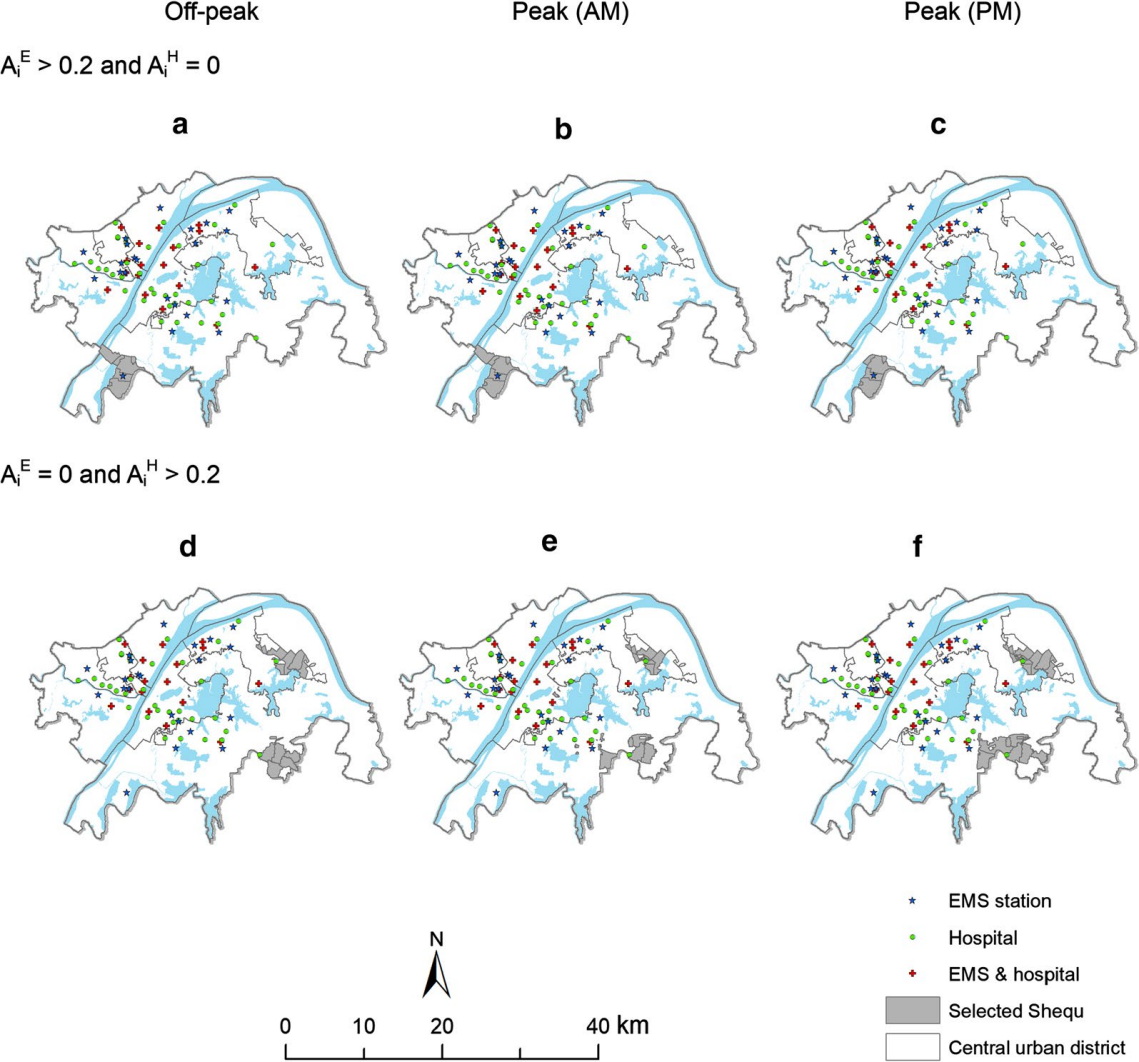
Locations of the Shequ with good access only in one single trip (${A}_{i}^{E}$ > 0.2 and ${A}_{i}^{H}$ = 0: **a** off-peak periods, **b** morning peak periods, **c** evening peak periods; ${A}_{i}^{E}$ = 0 and ${A}_{i}^{H}$ > 0.2: **d** off-peak periods, **e** morning peak periods, **f** evening peak periods)

Figure 9 highlights the shequs that have distinctly different access for the two single trips indicated by the E-2SFCA. That is, if a selected shequ in Fig. 9 has relatively good access in trip 1 with a score higher than 0.2 (see Fig. 7), it has relatively poor access in trip 2 with a score 0, and vice versa. Specifically, the five shequs that have good ambulance access but poor hospital access (i.e. more than 15 min away from the nearest hospital) are located mainly in the south of Hongshan. In particular, Fig. 9c indicates that there is one less shequ affected in the evening peak periods compared with off-peak (Fig. 9a) and morning peak periods (Fig. 9b). The shequs that have good hospital access but cannot be reached by an ambulance with 15 min are primarily located in the northeast of Qingshan and southeast of Hongshan. In this case, more shequs are affected: 14, 21 and 23 for the off-peak (Fig. 9d), morning (Fig. 9e) and evening (Fig. 9f) peak periods, respectively.

## Discussion

The results illustrate the impact of real traffic on EMS access and the differences in spatial patterns of ambulance and hospital access. Both methods show a marked decrease in EMS access during peak periods. About 73.9% of shequs can receive an ambulance or get to the nearest hospital within 10 min during off-peak periods, and this proportion decreases to about 45.5% for peak periods. The areas that are close to both sides of Yangtze River in the central parts of the study area are most affected by traffic. In general, the central areas have better ambulance, hospital and overall access than peripheral areas, particularly during off-peak periods. Most shequs with good ambulance access but poor hospital access are in the south of the study area.

The results highlight the importance of incorporating real traffic data as well as integrating both related trips (i.e. STI and TTI) in measuring access to EMS, which is supported by the empirical findings of previous studies using real EMS records. For example, the overall spatial accessibility of EMS is significantly reduced when traffic congestion is considered [17]. Regarding the significance of STI in EMS access and associated outcomes, it was estimated that the odds of patient survival with out-of-hospital cardiac arrest could be increased by 24% for a 1-min reduction of ambulance response time (O’Keeffe et al., 2001). Similarly, the probability of death in road accidents could be reduced by one third if the ambulance could arrive 10 min earlier [25]. In terms of the importance of and TTI, stroke victims should be transferred to hospitals as soon as possible to receive specialized medical treatment that is difficult to provide in the ambulance [3]. In the UK, historical data indicates that around half of the deaths caused by traffic crashes occurred at the scene or in the ambulance, that is, prior to receiving advanced medical treatment in definitive care locations [14].

In addition to the common findings with respect to the impact of peak periods, the two approaches, namely travel time and E-2SFCA, also suggest different spatial patterns in EMS access. For example, Fig. 5 indicates that most areas with better access are in the central parts of the study area and on both sides of Yangtze River. When incorporating the supply-to-population ratio, the results from E-2SFCA indicate that most areas with better access are located to the east of Yangtze River (see Fig. 8). The three districts in the west (i.e. Jiang’an, Jianghan and Qiaokou) have a large population, which might offset the advantages of having many surrounding EMS stations and hospitals. In contrast, although there is only one EMS station and one hospital to the east of Qingshan (see Fig. 2), the region has far fewer people, thus sharply increasing the likelihood of better access.

Considering the timeliness of rescue in emergencies, travel time is the most common indicator of EMS access. There are several advantages of using predicted travel times in measuring access to EMS. First, such measures can be obtained directly from online map services without the need of GIS software and preparing road network dataset. Second, the road network information provided by online map services is often more updated. Third, online map services usually account for real traffic conditions (e.g. road work or traffic congestion), which thus can offer more accurate travel time estimation. One major limitation of online map services, however, is that free calculation of predicted travel times is only available for a certain subset of origin–destination (OD) pairs, beyond which a payment must be made. For example, Google Maps offers 10,000 free OD calculations per month for each account and requires USD 5 for every additional set of 1,000 calculations. Comparatively, Baidu Map offers more free calculations–30,000 per day for each account and unlimited calculations per month for a fee of about USD 2800.

There are some limitations of this research. First, due to the lack of the real-world EMS run data, the residence was adopted in this research as the scene where patients receive an ambulance and then get transported to the hospital. As the central urban areas are often more densely populated with more health services (i.e. EMS stations and hospitals), it is not surprising that those areas have better access to EMS when only travel time is considered. However, it is common for emergencies to also occur at other places, such as highways or workplaces. Hence, it is necessary to consider additional scenes for EMS access. Second, the nearest hospital was used in measuring EMS access in trip 2. It should be noted that some diseases (e.g., trauma and stroke) can only be treated at specialized hospitals. Therefore, it would be useful to examine EMS access specifically with respect to specialized healthcare. Third, the travel time estimated by online map services might lack precision, which primarily depends on the type of path taken (small streets, avenues, highways). As the ambulances can overpass red lights and stop signs in hyper dense urban districts, the differences between predicted and actual times of arrival in such areas might be higher than those in peripheral urban/rural areas with unobstructed roads or highways. Forth, the potential access to EMS here does not consider PATI nor OSTI, which often varies depending on specific situations (e.g. types of diseases or severity of accidents). Finally, in addition to the peak and off-peak periods analysed above, it is possible that for the two trips (i.e. trip 1 and trip 2), one occurs during peak period and the other is in off-peak period, or vice versa, which again could be affected by the call and on-site rescue time.

Based on this research, some future work can be carried out to improve the EMS systems. First, the real-world EMS records, if available, can be utilized to validate the travel time estimated by online map services and improve the accuracy of STI and TTI prediction. In addition, the access measures can be improved by integrating PATI and OSTI if empirical data become available. Second, different on-scene time can be estimated from historical real-world EMS records for different types of diseases [28], which can be integrated with STI and TTI to obtain disease-specific access measures. Finally, empirical studies can be carried out to investigate how health outcomes such as mortality/morbidity is associated with access to EMS using proposed measures.

Regarding policy implications, first, the empirical results of spatial variations of ambulance and hospital access can serve as a reflection of public policies on EMS management and planning. For example, Figs. 5, 8 indicate that residents on the periphery of Hongshan district have relatively poor access to EMS and hospitals, and therefore additional health facilities can be sited in this area. Particularly, spatial optimization can be employed to identify the best spatial layout of EMS stations if health resources are limited so that ambulances can reach as many households as possible under given access constraint (i.e., 10 or 15 min for STI in trip 1) [27]. Accordingly, some of existing EMS stations can be relocated in order to improve emergency service access and efficiency. Likewise, similar approaches can be used to obtain the minimum number and locations of new EMS stations that are needed for a region if any scene is required to be reached by an ambulance from the nearest EMS station within certain time (i.e. STI) [37]. Second, effective measures are required to mitigate traffic congestion because EMS access obviously decreased during traffic peak hours. Third, between the two access measures employed here, travel time has the advantage of being easy to obtain with online map services and being straightforward to interpret. Therefore, it can be used in real-time decision-making relating to emergency services if health resources are sufficient and EMS stations have similar medical resources. Because it incorporates both supply and demand information, the E-2SFCA method is more suitable for long-term planning to respond to major accidents, hazards or pandemics. Finally, it should be noted that although the empirical study was carried out in Wuhan, China, both access measures employed in this research can be applied in other contexts as well. Predicted travel times are readily obtained by common online map services such as Google Maps in many parts of the world, and input parameters of the E-2SFCA that have been validated in diverse contexts are not unique to Wuhan (e.g. [7, 17]. Therefore, the work presented in this research can be done anywhere in the world with similar data sets to assess EMS access outside China.

## Conclusions

The EMS system is an important component of health services and plays a significant role in emergency and rescue services. Access as a primary indicator of EMS efficiency has been widely studied over the last few decades and many EMS access measures have been proposed. This paper expands the literature by demonstrating the importance of integrating the two related trips involved in EMS. Our empirical study focused on EMS access in Wuhan, China. In addition to the impact of peak traffic periods on EMS access, we found that good ambulance access does not necessarily guarantee good hospital access nor the overall access, and vice versa. The findings urge the need of accounting for both related trips in measuring EMS access as well as in planning EMS and hospital infrastructure.

## Data Availability

The datasets used and analysed during the current study are available from the corresponding author on reasonable request.
